# Integrated pharmacokinetics/pharmacodynamics parameters-based dosing guidelines of enrofloxacin in grass carp *Ctenopharyngodon idella* to minimize selection of drug resistance

**DOI:** 10.1186/1746-6148-9-126

**Published:** 2013-06-25

**Authors:** Lijuan Xu, Hao Wang, Xianle Yang, Liqun Lu

**Affiliations:** 1 Key Laboratory of Freshwater Fishery Germplasm Resources, Ministry of Agriculture, Shanghai Ocean University, Shanghai 201306, People's Republic of China

**Keywords:** Enrofloxacin, Grass carp *ctenopharyngodon idella*, Pharmacokinetics, Pharmacodynamics, *Aeromonas hydrophila*

## Abstract

**Background:**

Antibiotic resistance has become a serious global problem and is steadily increasing worldwide in almost every bacterial species treated with antibiotics. In aquaculture, the therapeutic options for the treatment of *A. hydrophila* infection were only limited to several antibiotics, which contributed for the fast-speed emergence of drug tolerance. Accordingly, the aim of this study was to establish a medication regimen to prevent drug resistant bacteria. To determine a rational therapeutic guideline, integrated pharmacodynamics and pharmacokinetics parameters were based to predict dose and dosage interval of enrofloxacin in grass carp *Ctenopharyngodon idella* infected by a field-isolated *A. hydrophila* strain*.*

**Results:**

The pathogenic *A. hydrophila* strain (AH10) in grass carp was identified and found to be sensitive to enrofloxacin. The mutant selection window (MSW) of enrofloxacin on isolate AH10 was determined to be 0.5 - 3 μg/mL based on the mutant prevention concentration (MPC) and minimum inhibitory concentration (MIC) value. By using high-performance liquid chromatography (HPLC) system, the Pharmacokinetic (PK) parameters of enrofloxacin and its metabolite ciprofloxacin in grass carp were monitored after a single oral gavage of 10, 20, 30 μg enrofloxacin per g body weight. Dosing of 30 μg/g resulted in serum maximum concentration (C_max_) of 7.151 μg/mL, and concentration in serum was above MPC till 24 h post the single dose. Once-daily dosing of 30 μg/g was determined to be the rational choice for controlling AH10 infection and preventing mutant selection in grass carp. Data of mean residue time (MRT) and body clearance (CLz) indicated that both enrofloxacin and its metabolite ciprofloxacin present similar eliminating rate and pattern in serum, muscle and liver. A withdraw time of more than 32 d was suggested based on the drug eliminating rate and pharmacokinetic model described by a polyexponential equation.

**Conclusions:**

Based on integrated PK/PD parameters (AUC/MIC, Cmax/MIC, and T>MPC), the results of this study established a principle, for the first time, on drawing accurate dosing guideline for pharmacotherapy against *A. hydrophila* strain (AH10) for prevention of drug-resistant mutants. Our approach in combining PK data with PD parameters (including MPC and MSW) was the new effort in aquaculture to face the challenge of drug resistance by drawing a specific dosage guideline of antibiotics.

## Background

Fluoroquinolones such as enrofloxacin and ciprofloxacin are widely used in the treatment of animal disease caused by both Gram-negative and Gram-positive bacteria [1]. The bactericidal activity of fluoroquinolones is concentration-dependent, thus the peak level (C_max_) and the amount of drug, as reflected by the area under the concentration-versus-time curve (AUC) in serum, are important predictors of the efficacy of fluoroquinolones [2]. Enrofloxacin, a fluoroquinolone carboxylic derivative, is Food and Drug Administration (FDA)-approved for treatment of individual pets and domestic animals in the United States. Enrofloxacin can be de-ethylated *in vivo* to its metabolite ciprofloxacin with a rate that is species-specific [3]. *Aeromonas hydrophila* (*A. hydrophila*) is a ubiquitous Gram-negative bacterium causing fatal hemorrhagic septicemia and dropsy in many commercially important freshwater fish worldwide [4].

*A. hydrophila* is also considered one of the major pathogens threatening the freshwater fish cultivation industry including grass carp *ctenopharyngodon idella*, the top aquaculture species of China [5]. Despite the severe economic loss, no vaccination against *A. hydrophila* infection has been commercially applied in China [6]. The main reason for the lack of commercial vaccines might be the existence of antigenic heterogeneity of various *A. hydrophila* strains in the field. Until now, antibacterial drugs through oral delivery are the popular and effective choice for the control of pandemics causing by *A. hydrophila*. In China, enrofloxacin is one of the effective antimicrobials in grass carp farms and has been widely applied in fish ponds nationwide for over 10 years [7].

Antibiotic resistance has become a serious global problem and is steadily increasing worldwide in almost every bacterial species treated with antibiotics [8]. In aquaculture, the therapeutic options for the treatment of *A. hydrophila* infection were only limited to several antibiotics, which contributed for the fast-speed emergence of drug tolerance [9]. Traditional one-for-all dosing guideline of enrofloxacin is not applicable any more. The failure to control bacterial disease in fish with antibiotics was most likely to result from sub-optimal therapy, irrespective of the degree of resistance. Enrofloxacin oral formulations have been commercialized in China. Farmers can use it to feed fish to treat bacterial infection. In fact, more and more pandemic *A. hydrophila* strains isolated from the diseased fish were with reduced susceptibility to enrofloxacin [10]. Given this, there is an urgent need to develop a medication regimen that prevents the formation of drug resistant bacteria.

Recently, new approaches were tested with an aim to increase efficacy of antibacterial drugs and to reduce resistance selection. It is well established that serum C_max_/MIC (ratio of maximum drug concentration to minimum inhibitory concentration) >8 and AUC/MIC (ratio of area under the concentration-time curve to minimum inhibitory concentration) >100 are required for efficient and optimal pharmacotherapy of enrofloxacin [11]. The mutant prevention concentration (MPC) is a new concept meant to face the increased prevalence of antibiotic resistance by using antibiotic concentrations able to prevent the selection of resistant bacteria populations [12]. Since T>MPC, exposure time of drug concentration above MPC, served as a more important factor in preventing drug resistance, AUC/MIC, C_max_/MIC and T>MPC were important integrated PK/PD parameters.

The aim of the present study was to develop a medication regimen against a disease caused by *A. hydrophila* strain (AH10) in grass carp by integrating pharmacodynamic (including MPC and MSW) and pharmacokinetic parameters for enrofloxacin. Although enrofloxacin is widely used as antimicrobial agent, its PK or PD parameters in grass carp are lacking. 10 ~ 30 μg/g is the dose of recommended in the industry [13]. To clarify the PK indices, oral dosing of 10, 20, and 30 μg/g body weight were individually examined in the study. The results of this study paved the way for establishing dosing guidelines to avoid selecting resistant mutants of specific fish bacterial pathogen with uncertain drug-susceptibility.

## Results and discussion

### The median lethal dose (LD_50_) of *A. hydrophila* (AH10) on grass carp

*A. hydrophila* was the most destructive bacterial pathogen of farmed grass carp. AH10 strain was isolated and identified according to the method of Roxana et al. [14] from diseased grass carp in 2011 (NCBI accession number: JX413114.1). The pathogen was stored in National Aquatic Pathogen Collection Center (No. 2011AH10). Grass carp was susceptible to laboratory infection with isolate AH10. External clinical signs of the infection included haemorrhaging and erratic swimming behavior. The seven day LD_50_ for grass carp by intraperitoneal route was 1.18 × 10^6^ CFU/mL (the 95% confidence interval was 5.53 × 10^5^ ~ 2.42 × 10^6^ CFU/mL), which was analyzed from the data in Table 1 by SPSS 16.0 software. Based on the clinical symptom and values of LD_50_, isolate AH10 should be regarded as a virulent strain.

**Table 1:** Infection of grass carp with AH10 by intraperitoneal route

**Pathogen amount (CFU/mL)**	**Fish amount**	**Dead number of fish**							**Accumulated dead number**
		**1d**	**2d**	**3d**	**4d**	**5d**	**6d**	**7d**	
2.5 × 10^3^	10	0	0	0	0	0	0	0	0
2.5 × 10^4^	10	0	0	1	1	0	0	0	2
2.5 × 10^5^	10	1	2	1	1	0	0	0	5
2.5 × 10^6^	10	2	3	1	2	0	0	0	8
2.5 × 10^7^	10	5	3	2					10
2.5 × 10^8^	10	10							10

### Activities of enrofloxacin on *A. hydrophila* (AH10) *in vitro*

The *in vitro* activities of enrofloxacin on isolate AH10 were subsequently investigated. The MIC value of enrofloxacin on isolate AH10 was 0.5 μg/mL, and that of quality control strain *A. hydrophila* ATCC 7966 was 0.25 μg/mL. The isolate AH10 was less sensitive to enrofloxacin than control strain ATCC7966. MPC values should be considered in drawing dosing strategies since traditional MIC-based dosing level might give rise to treatment failure due to the selection of drug-resistant mutant. The MPC of enrofloxacin on isolate AH10 was determined to be 6 MIC (3 μg/mL), which was above the resistance breakpoint for enrofloxacin (≥ 2 μg/mL). The MSW of enrofloxacin on isolate AH10 was determined to be 0.5 – 3.0 μg/mL, which reflected the difference between the measured MIC and MPC values. Existence of the MSW allowed us to predict the likelihood for resistance selection or prevention based on achievable and therapeutic drug concentrations [15]. Selective amplification of the non-susceptible AH10 population was expected to occur in the identified MSW of enrofloxacin. As far as we knew, this was the first study trying to apply MPC principles to optimize therapy and reduce resistance selection of fish bacterial pathogen.

**Figure 1:**
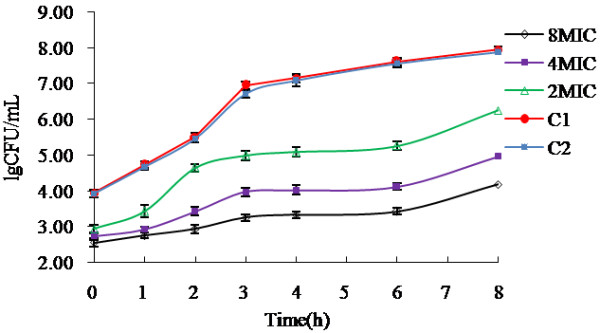
**Replication of isolate AH10 after 1 h exposure to enrofloxacin at indicated concentration.** Control groups (C1 and C2) referred to bacterial without drug treatment. (n = 3,The mean ± SD). After 1 h exposure to enrofloxacin with a concentration as indicated in the figure, the concentration of isolate AH10 was determined to be 8.71 × 10^3^ CFU/mL (C1), 8.51 × 10^3^ CFU/mL (C2), 8.71 × 10^2^ CFU/mL (2 MIC), 5.50 × 10^2^ CFU/mL (4 MIC) and 3.63 × 10^2^ CFU/mL (8MIC), respectively.

The post antibiotic effect (PAE) may contribute to the *in vivo* efficacy of enrofloxacin [16]. It was well known that enrofloxacin prevented the synthesis of bacterial DNA by gyrase, the PAE might represent the time for it to dissociate from the receptor binding sites and to diffuse out of the bacterium. It was generally believed that PAE was concentration dependent and directly related to the exposure time [17]. In this study, PAE of enrofloxacin was evaluated by exposure of isolate AH10 to it at 2, 4, and 8 times MIC for 1 h (Figure. 1). The PAE of enrofloxacin was 1.44 ± 0.36h, 1.57 ± 0.09h, 1.83 ± 0.21h, respectively. The data predicted that the dosing interval deduced from the time-concentration PK curve could be 1 – 2 h longer due to the existence of PAE. Although the *in vivo* PAE of enrofloxacin was still awaiting further investigation, our data at least suggested that the T>MPC seemed to be the dominant factor in drawing dose intervals since PAE of enrofloxacin was comparably short.

### Pharmacokinetics of enrofloxacin in grass carp

HPLC presents a simple, rapid, and reliable analytical method for detection and quantification of enrofloxacin and its metabolite ciprofloxacin. In our HPLC system, the limit of detection (LOD) for enrofloxacin and ciprofloxacin was 0.02 μg/mL and 0.004 μg/mL, respectively. The sample concentration was calculated by comparison of its peak area with the peak area of a nominal concentration of an external standard. The chromatography area and the concentration of enrofloxacin and ciprofloxacin showed a linear relationship. The standard curve equation of enrofloxacin is y = 191.7× - 52.68 (r = 0.9995), and the standard curve equation of ciprofloxacin is y = 92.74× – 12.00 (r = 0.9999). The standard was routinely corrected by the concentration factor and the recovery rate [18]. The linearity of our method was confirmed using the classical tests by analysis of variance.

**Figure 2:**
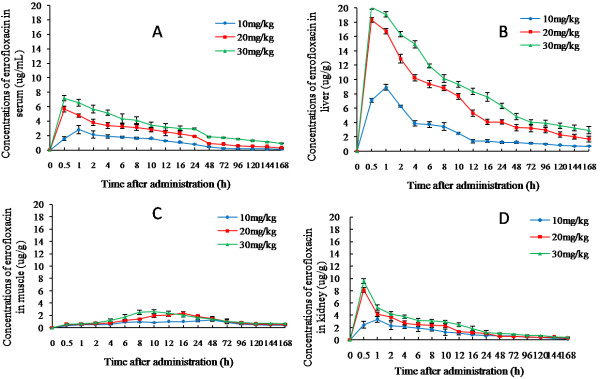
**Enrofloxacin concentration-time curves in serum and tissues of grass carp at different administration doses.** Standard deviation is given at vertical bars (n = 5, The mean ± SD). At three doses of enrofloxacin, the C_max_ of enrofloxacin in liver is highest and the C_max_ of enrofloxacin in muscle is lowest. The time to C_max_ in muscle for enrofloxacin was significantly longer than in serum and other tissues. (**A**: Serum; **B**: Liver; **C**: Muscle **D**: Kidney).

The concentration-time profiles of serum and tissue after an oral dose of 10 μg/g, 20 μg/g, and 30 μg/g were separately shown in Figure 2. In general, high dose (20 μg/g and 30 μg/g) resulted in a shorter time (0.5 h) to peak level than lower dose (1 h for dose of 10 μg/g), and the time to reach the peak level in muscle (≥10 h) was much longer than serum & other tissues for all the three doses. 24 h post drug delivery, serum concentration of enrofloxacin was 0.801 μg/mL, 1.918 μg/mL, and 2.953 μg/mL, respectively. The low elimination rate generally predicted a positive clinical result and correlated with the extensive application of enrofloxacin in controlling bacterial disease in grass carp. Peak levels (C_max_) of liver (8.941 μg/g, 18.344 μg/g, and 20.076 μg/g, respectively) was the highest, followed by kidney (3.362 μg/g, 8.152 μg/g, and 9.587 μg/g), serum (2.837 μg/g, 5.685 μg/g, and 7.150 μg/g), and muscle (1.320 μg/g, 2.369 μg/g, and 2.628 μg/g). These data were basically in consistence with PK parameters of enrofloxacin in other fish species [19].

All the data were analysed by Kinetica4.4, the results showed that it was a two-compartment model with zero absorption in serum, liver and kidney, a two-compartment model with first-order absorption in muscle (Table 2). The correlation coefficient exceeded 0.99 and high model criteria (>5) were obtained. Listed PK parameters were demonstrated in Table 3. It was worth to note that model-independent parameters such as elimination half-life (t_1/2_), area under the curve (AUC), and clearance (CLs) were calculated for each of the five fish and averaged in the table.

**Table 2:** Best concentration-time equations of enrofloxacin in grass carp

**Dose**	**Tissues**	**Equation**
10 μg/g	Serum	C = 0.844e^-0.114t^ + 0.500e^-0.016t^
	Liver	C = 3.007e^-0.087t^ + 1.634e^-0.005t^
	Muscel	C = 3.334e^-0.022t^ + 1.025e^-0.159t^ – 4.359e^-0.2656t^
	Kidney	C = 1.109e^-0.027t^ + 0.164e^-0.025t^
20 μg/g	Serum	C = 1.707e^-0.166t^ + 1.600e^-0.015t^
	Liver	C = 9.072e^-0.191t^ + 4.631e^-0.015t^
	Muscel	C = 4.630e^-0.055t^ + 0.758e^-0.003t^ – 5.388e^-0.216t^
	Kidney	C = 7.39859e^-0.0244547t^ + 4.01081e^-0.0626125t^
30 μg/g	Serum	C = 3.904e^-0.164t^ + 3.013e^-0.007t^
	Liver	C = 14.577e^-0.066t^ + 5.887e^-0.004t^
	Muscel	C = 2.269e^-0.066t^ + 1.365e^-0.005t^ – 3.634e^-0.211t^
	Kidney	C = 6.154e^-0.044t^ + 4.044e^-0.036t^

**Table 3:** PK parameters of enrofloxacin in grass carp (n = 5)

**Tissues Parameters**	**Dose**											
	**10 mg/kg**				**20 mg/kg**				**30 mg/kg**			
	**Serum**	**Liver**	**Muscle**	**Kidney**	**Serum**	**Liver**	**Muscle**	**Kidney**	**Serum**	**Liver**	**Muscle**	**Kidney**
A(μg/mL)	0.844	3.007	3.334	1.109	1.707	9.072	4.630	7.162	3.904	14.577	2.269	6.154
B(μg/mL)	0.500	1.634	1.025	0.164	1.6	4.631	0.758	3.682	3.013	5.887	1.365	4.044
A(/h)	0.114	0.087	0.022	0.027	0.166	0.191	0.055	0.024	0.164	0.068	0.066	0.044
B(/h)	0.016	0.005	0.159	0.025	0.015	0.015	0.003	0.063	0.007	0.004	0.005	0.036
Ka(/h)	0.491	0.275	0.266	0.478	0.671	1.787	0.216	0.764	0.502	1.445	0.211	0.676
Kel(/h)	0.037	0.020	0.013	0.027	0.024	0.019	0.160	0.024	0.015	0.013	0.143	0.024
T_1/2α_(h)	6.098	0.080	3.126	25.938	4.186	36.262	12.595	28.344	4.348	10.145	10.515	27.775
T_1/2β_(h)	42.728	86.253	95.070	42.375	46.757	110.452	150.884	107.861	97.363	184.996	161.381	124.271
Tmax(h)	1.000	1.000	48.000	1.000	0.500	0.500	16.000	0.500	0.500	0.500	10.000	0.500
MRT_0-t_(h)	44.566	62.765	63.938	55.871	51.841	63.093	67.096	56.620	144.573	65.005	68.159	56.620
Cmax (μg/mL)	2.837	8.941	1.320	3.362	5.685	18.344	2.369	8.152	7.151	20.076	2.628	9.587
AUC_24_ (μg/mL·h)	25.838	66.108	21.683	33.183	67.585	114.168	40.349	49.589	90.486	242.313	44.666	66.359
AUC (μg/mL·h)	72.538	210.118	129.154	102.899	171.984	338.316	175.160	127.511	318.187	425.763	188.914	181.293
CLs (L/kg·h)	0.155	0.030	0.054	0.080	0.100	0.029	0.095	0.108	0.067	0.020	0.124	0.122

A(μg/mL), Intercept of the linear equation on log transformed data; B(μg/mL), Slope of the linear equation on log transformed data; α(/h), Distribution rate constant; β(/h), Elimination rate constant; Ka(/h), Absorption rate constant; Kel(/h), elimination rate constant from the central compartment; T1/2α(h), Half-life of distribution; T_1/2β_(h), Half-life of elimination; Tmax(h), Time required to reach Cmax in the interval; MRT_0-t_(h), Mean residue time of drug in body from zero to 168h; Cmax(μg/mL), Maximum concentration in the interval; AUC_24_(μg/mL·h), Area under the drug concentration-time curve from the time zero to 24h; AUC(μg/mL·h), Area under the drug concentration-time curve from the time zero to last time point; CLs(L/kg·h), total body clearance.

The serum concentration was the key factor for drug effectivity. At the tested oral dose of 10 μg/g, 20 μg/g and 30 μg/g in three independent experiments, the distribution half life (T_1/2α_) of enrofloxacin in serum was 6.098 h, 4.186 h, and 4.348 h, respectively. AUC of enrofloxacin in serum was 72.538 μg/mL·h, 171.984 μg/mL·h, and 318.187 μg/mL·h, respectively. These data suggested that higher dose resulted in significant higher AUC value or clinical result than lower dose, but with a similar absorption rate. The muscle concentration might be important in deciding the withdrawal time. The terminal (elimination) half life (T_1/2β_) of enrofloxacin in muscle was 95.070 h, 150.884 h, and 161.381 h, respectively. The AUC value of enrofloxacin in muscle was 129.154 μg/mL·h, 175.160 μg/mL·h, and 188.914 μg/mL·h, respectively. These results implied that the elimination rate in muscle was low and seemed independent of the dose level at higher dose (20 μg/g and 30 μg/g), and the similar AUC value of the two dosal levels further suggested that the withdrawal time might be similar, which was ideal for disease and drug-residual control since better clinical result might be achieved by increasing the dose level while keeping a similar withdrawal time.

### Pharmacokinetics of metabolite ciprofloxacin in grass carp

Although this study was aimed to examining potential dosage regimens for enrofloxacin against a specific bacterial pathogen, the PK parameters of its major metabolite ciprofloxacin was investigated in parallel with the analysis of enrofloxacin. It was well accepted that the rate of transformation of enrofloxacin to ciprofloxacin in aquatic animals was significantly less than that in terrestrial animals [3], which suggested that enrofloxacin was mainly responsible for clinical efficacy in fish. However, the marker residue for tissues after oral dose of enrofloxacin was the sum of the enrofloxacin and ciprofloxacin residues. Although ciprofloxacin itself was forbidden to be used in aquatic species, the permitted residue level of ciprofloxacin in fish was 0.030-0.050 μg/g in most countries. Thus, PK parameters of ciprofloxacin would be helpful in determining a suitable withdrawal time for enrofloxacin in grass carp.

**Figure 3:**
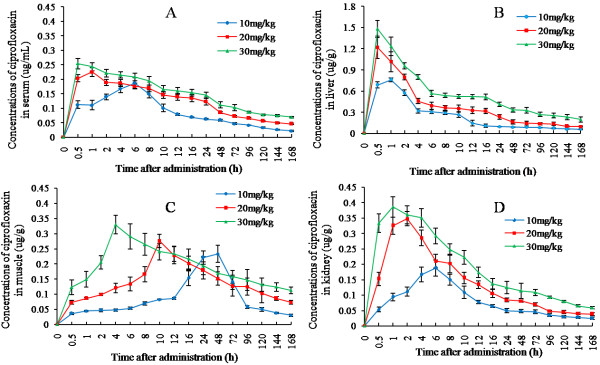
**Ciprofloxacin concentration-time curves in serum and tissues of grass carp at different administration doses of enrofloxacin.** Standard deviation is given at vertical bars. (n = 5, The mean ± SD). At three doses of enrofloxacin, the C_max_ of ciprofloxacin in liver is highest and the C_max_ of ciprofloxacin in serum is lowest. Similar to enrofloxacin, the time to C_max_ in muscle for ciprofloxacin was significantly longer than in serum and other tissues. (**A**: Serum; **B**: Liver; **C**: Muscle **D**: Kidney).

Figure 3 depicted the time course of metabolite ciprofloxacin concentrations in serum, liver, kidney, and muscle from grass carp after oral gavage of enrofloxacin at a dose of 10 μg/g, 20 μg/g, and 30 μg/g, respectively. In general, ciprofloxacin was detectable 0.5 h after drug delivery and high dose resulted a shorter time to reach C_max_. As expected, only a small portion of enrofloxacin was transmitted to ciprofloxacin. At the dose of 30 μg/g of enrofloxacin, the C_max_ of ciprofloxacin in serum, liver, muscle, and kidney was only 0.254 μg/mL, 1.480 μg/g, 0.329 μg/g, and 0.386 μg/g, respectively. Similar to enrofloxacin, the time to C_max_ in muscle for ciprofloxacin was significantly longer than in serum and other tissues. For example, the T_max_ for serum, liver, kidney, and muscle at the dose of 30 μg/g was 0.5 h, 0.5 h, 1 h and 4 h, respectively.

Analyzed by Kinetica4.4, the concentration-time profiles of ciprofloxacin could be described by a one-compartment model (Table 4). The correlation coefficient exceeded 0.99 and high model criteria (>5) were obtained.

**Table 4:** Best concentration-time equations of ciprofloxacin in grass carp

**Dose**	**Tissues**	**Equation**
10 μg/g	Serum	C = 0.119e^-0.010t^ + 0.119e^-0.078t^
	Liver	C = 0.178e^-0.008t^ + 0.178e^-0.249t^
	Muscel	C = 0.048e^-0.001t^ + 0.048e^-0.171t^
	Kidney	C = 0.125e^-0.011t^ + 0.125e^-0.202t^
20 μg/g	Serum	C = 0.175e^-0.009t^ + 0.175e^-0.268t^
	Liver	C = 0.451e^-0.008t^ + 0.451e^-0.222t^
	Muscel	C = 0.188e^-0.003t^ + 0.188e^-0.171t^
	Kidney	C = 0.222e^-0.012t^ + 0.222e^-0.214t^
30 μg/g	Serum	C =0.209e^-0.007t^ + 0.209e^-0.248t^
	Liver	C = 0.710e^-0.007t^ + 0.710e^-0.217t^
	Muscel	C = 0.260e^-0.005t^ + 0.260e^-0.222t^
	Kidney	C = 0.488e^-0.015t^ + 0.488e^-0.211t^

Listed PK parameters were demonstrated in Table 5. By comparison of the AUC of enrofloxacin and ciprofloxacin at different dosage (10 μg/g, 20 μg/g, 30 μg/g), the transformation rate of enrofloxacin to ciprofloxacin in serum was determined to be 0.85%, 1.13% and 1.87%, respectively. In liver, the rate was 4.93%, 6.09% and 7.59%, respectively; in kidney, the rate was 1.29%, 1.74%, and 1.88%, respectively; in muscle, the rate was 1.10%, 1.38%, and 2.33%, respectively. Although ciprofloxacin demonstrated a similar residue time (MRT_0-t_) than enrofloxacin, the CLs of ciprofloxacin was higher than enrofloxacin at all the three doses, which suggested that the withdrawal time could be decided according to the time to reach zero residue level of enrofloxacin since the elimination rate of ciprofloxacin was faster.

**Table 5:** PK parameters of ciprofloxacin in grass carp (n = 5)

**Tissues Parameter**	**Dose**											
	**10 mg/kg**				**20 mg/kg**				**30 mg/kg**			
	**Serum**	**Liver**	**Muscle**	**Kidney**	**Serum**	**Liver**	**Muscle**	**Kidney**	**Serum**	**Liver**	**Muscle**	**Kidney**
A(μg/mL)	0.104	0.172	0.048	0.118	0.169	0.435	0.185	0.21	0.203	0.687	0.254	0.453
A(/h)	0.01	0.008	0.001	0.011	0.009	0.008	0.003	0.012	0.007	0.007	0.005	0.015
Ka(/h)	0.078	0.249	0.171	0.202	0.268	0.222	0.171	0.214	0.248	0.217	0.222	0.211
Kel(/h)	0.031	0.027	0.016	0.019	0.02	0.028	0.012	0.022	0.019	0.025	0.012	0.023
T_1/2α_(h)	70.300	54.558	126.553	46.273	74.727	74.283	129.297	57.892	92.733	98.544	140.684	63.202
Tmax(h)	6.000	1.000	48.000	6.000	1.000	0.500	10.000	2.000	0.500	0.500	4.000	1.000
Cmax(μg/mL)	0.187	0.759	0.233	0.188	0.225	1.219	0.276	0.348	0.254	1.480	0.329	0.386
MRT_0-t_(h)	61.453	61.61	60.652	59.982	64.912	62.200	71.581	63.192	69.093	67.674	73.703	64.946
AUC(μg/mL·h)	8.392	17.09	2.704	7.633	13.516	29.941	21.380	12.166	17.665	55.732	26.675	18.329
CLs(L/kg·h)	0.920	0.429	17.893	0.913	1.078	0.214	0.272	0.572	1.072	0.102	0.210	0.331

A(μg/mL), Intercept of the linear equation on log transformed data; α(/h), Distribution rate constant; Ka(/h), Absorption rate constant; Kel(/h), elimination rate constant from the central compartment; T_1/2α_(h), Half-life of distribution; Tmax(h), Time required to reach Cmax in the interval; Cmax(μg/mL), Maximum concentration in the interval; MRT_0-t_(h), Mean residue time of drug in body from zero to 168h; AUC(μg/mL·h), Area under the drug concentration-time curve from the time zero to last time point; CLs(L/kg·h), total body clearance.

### Dosing guidelines of enrofloxacin based on integrated PK/PD parameters

**Figure 4:**
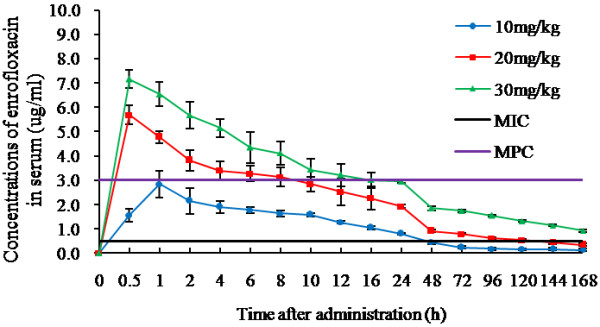
**Dtermination of T>MPC at the indicated dosing levels (n = 5, The mean±SD).** The T>MPC for the 10 μg/g dosage was 0 h, the T>MPC for the 20 μg/g dosage was 10 h, while T>MPC for the 30 μg/g dosage could reach 24 h.

Overuse and misuse of antimicrobial drugs had favoured the growth of resistant organisms. Inappropriate dosage regments included misuses in dose, dosage interval, duration of treatment, route and conditions of administration. In this study, we focused on dose and dosage interval selection. Traditional PK/PD indices (AUC/MIC, T > MIC, and C_max_/MIC) have been proposed to predict the success or failure of therapy, which only considered one PD biomarker, MIC. Although positive clinical outcome might be achieved by a dosage regimen based on these indices, it didn’t rule out the possibility of selecting drug-resistant mutant. Challenged by the emergence and spread of various resistance strains worldwide, the concepts of MSW and MPC provided new conceptual basis for PK/PD approaches in deciding dosing guidelines: treatments should maximize the time during which enrofloxacin cocentrations at the site of infection were above the MPC, and minimize the time during which these concentrations were in the MSW. Thus T>PC, instead of of T>MIC, was considered the most important factor in deciding dose regimens for the prevention of mutant selection. Although tissue drug concentration demonstrated difference with serum, serum concentration was more relevant in predicting antibiotic efficacy. Thus, serum PK/PD parameters were the major data that was based to draw a dosing guideline here (Table 6 and Figure 4).

**Table 6:** Serum PK-PD parameters

**Dosage(μg/g)**	**Cmax/MIC**	**AUC**_**24**_**/MIC**	**MPC(ug/ml)**	**MSW(ug/ml)**
10	5.68	51.68	3.0	0.5 ~ 3.0
20	11.37	135.17	3.0	0.5 ~ 3.0
30	14.30	180.97	3.0	0.5 ~ 3.0

In general, AUC_24_/MIC > 100 and C_max_/MIC > 8 predicted a clinical outcome of enrofloxacin [11]. In our analysis, AUC_24_/MIC and C_max_/MIC of enrofloxacin in serum were 51.68 and 5.68 at the dosage of 10 μg/g, respectively; 135.17 and 11.37 at the dosage of 20 μg/g, respectively; 180.97 and 14.30 at the dosage of 30 μg/g, respectively (Table 6). The data suggested that a dosage more than 20 μg/g might achieve good enough therapeutic result. However, the T>MPC for the 20 μg/g dosage was only 10 h, while T>MPC for the 30 μg/g dosage could reach 24 h. To prevent selection of resistant mutant, the dosage interval for the level of 20 μg/g was 10 h, which suggested a twice-daily dose. In contrast to it, once-daily dose of 30 μg/g was adequate for the maximum time of enrofloxacin concentration above MPC. Apparently, dosage level of 30 μg/g was more cost-effective. PAE of enrofloxacin at both these two dosages were 1–2 h in our assay, thus PAE wouldn’t significantly affect the choice of dosage interval for enrofloxacin in grass carp.

Elisa R et al. [20] suggested that the efficacy of administering enrofloxacin at 10 mg/kg in medicated water to turkeys was evaluated by applying a PK/PD approach to the kinetic parameters obtained after oral pulsed administration and to the MIC values of avian pathogenic *Escherichia coli* (APEC) strains isolated from commercial turkey flocks. The results were different from ours because of different tested animal species and pathogens, more importantly due to the different MIC values. Study on PK-PD indices of enrofloxacin in *Escherichia coli* O78/H12 infected chickens [21] also showed that PK-PD studies should include not only AUC/MIC and Cmax/MIC estimation but also determination of MPC values, which could describe better response of bacterial strain to the used fluoroquinolones. The high dose (2.5 ml enrofloxacin/1 L drinking water) of enrofloxacin was better than low dose (0.5 ml enrofloxacin solution/1 L drinking water) for efficient eradication of bacteria.

Reasonable dosage regimen should be based on PK/PD studies and determined by practical application of the test animals. The size of the dose and dosing interval were the key indices to meet the requirement of positive clinical outcome of fish: enough time of *in vivo* therapeutic concentrations, no adverse effects on the body, and acceptable withdrawal period. Usually concentration-dependent antibacterial drugs, like enrofloxacin, should be delivered through once-daily dosing approach, which was crucial for higher C_max_ or C_max_/MIC ratio to maximize the opportunity to get the best bactericidal action and clinical effect. The fact that Gram-negative bacteria became temporarily and reversibly adaptive resistant 2 h after administration of antimicrobial drugs also suggested that longer dosing interval could reduce the risk for development of adaptive resistance by microorganisms [22]. Thus, Once-daily drug delivery also contributed to maximize the bacterialcidal effect of enrofloxacin by avoiding adaptive resistance.

### Recommended withdrawal time based on the elimination rate of enrofloxacin and ciprofloxacin

Many countries protected consumer health from possible drug residues in fish by setting tolerable concentrations of drug residues, and surveillance agencies had been checking for compliance with the relevant directives. To ensure delivery of safe fish products to consumers, the withdrawal time of drugs must be respected [23]. It was worth to mention that the elimination rate of both enrofloxacin and ciprofloxacin was slowest in muscle. Thus, the muscle residue levels apparently represented the length of withdrawal period. From the concentration-time equations of both enrofloxacin and ciprofloxacin at a dosage of 30 μg/g in muscle (Table 2 and 4), it would take at least 32 d for enrofloxacin (32 d) and ciprofloxacin (15 d) to be depleted to a permitted residue level of 30 μg/kg. According to the present data, the proposed withdrawal period in grass carp should not be less than 32 d. Both enrofloxacin and ciprofloxacin levels reached to the detection limit of our HPLC system at 32 d after the dosage of 30 μg/g, and became undetectable at 37 d post drug delivery. This result partially validated our PK/PD parameters. However, the present study didn’t consider the effect of continuous drug delivery (generally 3–7 d for bacterial disease) on the elimination rate of enrofloxacin or ciprofloxacin, a withdrawal period of more than 32 d might be expected.

## Conclusions

The PD parameters (MIC, MPC, and MSW) of *A. hydrophila* AH10, isolated from grass carp *Ctenopharyngodon idella*, were characterized. The PK parameters in serum and tissues (liver, kidney, and muscle) of enrofloxacin following a single oral treatment in grass carp *Ctenopharyngodon idella* at three different dosage levels (10, 20, and 30 μg/g) were investigated. Integrated PK/PD parameters (AUC/MIC, C_max_/MIC, and T>MPC) suggested that once-daily dosage of 30 μg/g predicted a positive clinical outcome and minimize the selection of drug-resistant AH10 mutant. Our approach in combining PK data with PD parameters (including MPC and MSW) was the new effort in aquaculture to face the challenge of drug resistance by drawing a specific dosage guideline of antibiotics.

## Methods

### Fish

All animal procedures were reviewed and approved by the Institutional Animal Care and Use Committee at Shanghai Ocean University. Animal care and experimentation were undertaken in accordance with Ocean University of Shanghai animal care guidelines. 500 grass carp *Ctenopharyngodon idellas* (body weight of around 200 g) were obtained from a fish hatchery in Shanghai, China and maintained in 600-L fiberglass tanks with circulating, filtered and well-aerated tap water at 22.0 ± 1.0°C under a 12:12 h day:night cycle and were reared under normoxia (7.0 ± 0.2 mg O_2_/L) for 2 week prior to experimentation, and with no history of fluoroquinolone treatment. The fish were being fed daily with artificial feed (Charoen Pokphand Group).

### Chemicals

Enrofloxacin and ciprofloxacin powder standards were obtained from Sigma (St Louis, MO, USA). Prototype enrofloxacin (≥99%) was supplied from Zhejiang Xinchang Pharmaceutical Co., Ltd. Organic solvents used in this study were of HPLC grade (Sigma-Aldrich, China). other chemicals were analytical grade (Dingguo Biotech, Beijing); Mueller-Hinton Broth (MHB) and Mueller-Hinton Agar (MHA) was supplied from Sinopharm Chemical Reagent Beijing Co., Ltd.

### Drug administration

In oral gavages, the fish were divided into three groups (300, 100/group), fish were given a dose of 10, 20, 30 μg/g body weight individually. Enrofloxacin solution (60 g/L) was forced into the stomach of the grass carp using a 1-ml syringe fitted with an obtuse 7^#^ gauge needle. (225 fish were killed after the end of the experiment.)

### Sample collection

Five live fish were sampled at intervals ranging from 0.5-168 h after the administration. Fish were anesthetized with 2-phenoxyethanol (2 mL/L) before handling. One milliliter of blood was drawn into heparinized syringes from the caudal artery. Blood samples were centrifuged at 10000 × g for 10 min, the serum was then collected and stored at −20°C. Fish were than euthanized by concussion. Liver, kidney, and muscle tissues were taken and frozen at −20°C.

### Analytical methods

#### HPLC (high performance liquid chromatography)

Serum and tissue concentrations of enrofloxacin and ciprofloxacin were assayed by HPLC. HPLC analysis was performed with Agilent 1100 consisted of double pump, auto-injector, column temperature tank, and fluorescence detector that was set at 280 and 450 nm as excitation and emission wavelengths, respectively. The mobile phase consisted of acetonitrile and 0.01 M tetrabutylammonium bromide solution (5:95 V/V). The pH of tetrabutylammonium bromide solution was adjusted to 3.1 by phosphoric acid. Zorbax SB C-18 column (4.6 × 150 mm) was used for the separation. The flow rate was 1.0 ml/min. The column temperature was controlled at 40°C. External standards were enrofloxacin and ciprofloxacin (Sigma-Aldrich, China).

Extraction of the compounds from serum and tissue samples were achieved with 90% acidified acetonitrile (containing 1% acetic acid) and 10% 0.15M hydrochloric acid according to a modified protocol [3]. Recovery and precision of the method, as well as the standard calibration curves, were determined by a routine method [24]. For both enrofloxacin and ciprofloxacin, the assay was linear between 0.01 and 10 μg/ml or μg/mg.

### LD_50_

In order to estimate the median lethal dose 50% (LD_50_) of isolate AH10, duplicated groups of grass carp of 100–120 g (50, 10/group) were injected intraperitoneally with 200 μl of serial dilutions of isolate AH10, from 1 × 10^3^ to 1 × 10^8^ CFU/ml. Control groups were injected with PBS. Mortalities were recorded for 7 days. (35 fish were killed after the end of the experiment.)

### MIC

Minimum inhibitory concentration (MIC) was determined by the double-tube method [25,26]. Briefly, 1 ml of 128 μg/mL enrofloxacin was dissolved in 1 ml fresh Mueller-Hinton Broth (MHB) media in tube #1. Subsequently, 1 ml of culture from tube #1 was removed and added to tube #2 containing 1 ml MHB media. The series of dilutions was carried out for a total of 12 tubes. All 12 testing tubes along with 2 control tubes were separately inoculated with 20 μL (10^6^ CFU/mL) isolate AH10 suspension and incubated in a 30°C (the optimum growth temperature of A. hydrophila strain is 28 ~ 30°C) incubator for 24 h. The lowest drug concentration among tubes with no bacterial growth would be the drug’s minimum inhibitory concentration (MIC). This experiment was performed in 3 replicates. Reference strain *A. hydrophila strain* ATCC 7966 (store in Key Laboratory of Freshwater Fishery Germplasm Resources, Shanghai) was used for quality control.

### MPC and MSW

The mutant prevention concentration (MPC) indicated the susceptibility of the small number of resistant mutant bacterial present before any drug treatment. The MPC was determined by methods described by Frederique Pasguali et al. [27]. Briefly, isolate AH10 was cultured in MHB media for 24 h. Then this cultured suspension was centrifuged (at 4000 × g for 10 min) and used MHB media to resuspend the isolate AH10 to a concentration of 10^10^ CFU/mL. 300 μL isolate AH10 suspension, containing more than 10^10^ CFU/mL, were plated on each of four Mueller-Hinton Agar (MHA) plates supplemented with either enrofloxacin at concentration equal to 1×, 2×, 3×, 4×, 5×, 6×, 7×, 8×, 9×, 10× MIC. Plates were incubated at 30°C for 48h, colonies counted and incubated again for an additional 72h. The MPC was recorded as the lowest drug concentration preventing the emergence of any mutant after 48h and 120h incubation. Each experiment was carried out three times.

### PAE

Post antibiotic effect (PAE) was classically defined as the period of bacterial growth suppression that persisted after a limited exposure of organisms to antimicrobials. In establishing dosing schedule, duration of PAE should be added to the effective therapeutic period, which was drawn from the time window with a concentration more than MIC or MPC. The presence of PAE was determined by methods described by Gudmundsson [28]. To assess PAE, isolate AH10 (2.5 × 10^3^ CFU/ml) was exposed to enrofloxacin at 2 – 8 MIC for 1 h. At the end of the exposure period, the antibiotic was removed by diluting 1: 10^3^ into the same prewarmed medium. For quantification of the PAE, viable counts were determined before and after drug exposure, and then hourly for 12 h. PAE was expressed in hour and determined by the formula PAE = T-C. T represented the time for the drug-treated inoculum to amplify to a concentration of 10 folds of the starting point, C represented the time for the untreated control culture to increase to 10 folds of its starting point.

### Pharmacokinetic analysis

The computer program Kinetica4.4 (Thermo Fisher Scientific) was used to derive pharmacokinetic values, which described the drug concentration-time data very well. The pharmacokinetic parameters were analyzed based on statistical moment theory for the serum and tissues concentration-time data of enrofloxacin and its active metabolite ciprofloxacin. The following PK parameters were determined and analyzed: C_max_ (peak concentration), T_1/2α_ (half-life of the absorption rate constant), T_1/2β_ (half-life of the elimination rate constant), AUC_24_ (area under the concentration-time curve from time zero to 24 h), MRT_0-t_ (mean residue time in body from zero to 168 h), and CLs (total body clearance).

### Pharmacodynamics analysis

Due to the determined low transformation rate (less than 5%) of enrofloxacin to ciprofloxacin in this study, the antibiotic function of ciprofloxacin was found insignificant as a metabolite. Pharmacodynamics analysis was only performed on enrofloxacin. By integrating experimental pharmacokinetic data and *in vitro* activities of enrofloxacin on isolate AH10, optimal microbiological outcomes were most likely predicted by C_max_/MIC, AUC/MIC, and T>MPC.

## Competing interests

The authors declare that they have no competing interests.

## Authors’ contributions

LJX and LQL conceived the study, participated in its design and coordination, data acquisition and analysis, and manuscript elaboration. HW participated in data acquisition and carried out the Pharmacokinetic analysis. XLY participated in the study design and coordination, and helped with the interpretation of data. All authors read and approved final manuscript.
